# The epidemiology of amoebiasis in Thi-Qar Province, Iraq (2015-2020): differentiation of *Entamoeba histolytica* and *Entamoeba dispar* using nested and real-time polymerase chain reaction

**DOI:** 10.4178/epih.e2021034

**Published:** 2021-05-06

**Authors:** Mohammed Hassan Flaih, Ruaa Majid Khazaal, Manar Karem Kadhim, Khwam Reissan Hussein, Falah Abd Bashir Alhamadani

**Affiliations:** 1 Department of Medical Laboratory Techniques, Nasiriyah Technical Institute, Southern Technical University, Nasiriyah, Iraq; 2 Thi-Qar Education Directorate, Nasiriyah, Iraq; 3 Bint Al-Huda Maternity and Children Teaching Hospital, Thi-Qar Health Office, Nasiriyah, Iraq

**Keywords:** Intestinal diseases, Polymerase chain reaction, *rRNA* gene, *Entamoeba histolytica*, *Entamoeba dispar*

## Abstract

**OBJECTIVES:**

The objective of this study was to evaluate the present status of amoebiasis in Thi-Qar Province in southern Iraq, and to determine the presence of *Entamoeba histolytica* and *Entamoeba dispar* with nested and real-time polymerase chain reaction (PCR).

**METHODS:**

Epidemiological data were obtained from the public health department of the Thi-Qar Health Office (2015-2020). Eighty stool samples were also randomly collected from patients ≤12 year of age with diarrhea at 2 hospitals between the beginning of February 2020 and the end of October 2020. These samples were selected after microscopy to identify the *18S rRNA* gene in *Entamoeba* DNA.

**RESULTS:**

Of the 341,554 cases of intestinal parasitic infections, 38,004 (11.1%) individuals were recorded as having amoebiasis, which accounted for the highest proportion of infections in 2015 (26.1%) and the lowest in 2020 (8.1%). Amoebiasis was distributed among all age groups, with the age group of 5-14 years accounting for the highest proportion (27.3%). In molecular testing, 42 (52.5%) out of 80 samples were positive for the *18S rRNA* gene (888 bp). Using nested PCR, *E. histolytica* (439 bp) was detected in 25 (31.3%) samples and *E. dispar* (174 bp) in 14 (17.5%), while using real-time PCR, *E. histolytica* and *E. dispar* were detected in 28 (35.0%) and 15 (18.8%) samples, respectively.

**CONCLUSIONS:**

Epidemiological data confirmed that amoebiasis is endemic in this province, and is not limited to certain months. Our study confirms the applicability of molecular identification to detect pathogenic and non-pathogenic *Entamoeba* to prescribe the appropriate drug.

## INTRODUCTION

Amoebiasis is still considered to be a global health problem that spreads in tropical and subtropical regions [1]. The transmission of amoebiasis in developing countries is usually due to poor sanitation, poor hygiene, and crowded living conditions, whereas it is mostly transmitted in developed countries by people who travel from endemic countries [2,3]. About 500 million people are believed to be with amoebiasis worldwide [4]. Previous studies have estimated that 50 million people contract amoebiasis annually and that 100,000 people die from amoebiasis every year [5,6]. However, amoebiasis is considered the third-highest cause of death among human parasitic infections [7,8].

Although the genus *Entamoeba* contains six species (*E. coli, E. histolytica, E. dispar. E. moshkovskii, E. bangladeshi, E. hartmanni*, and *E. poleki*) that colonize the human large intestine, only *E. histolytica* is considered a pathogen that invades the intestinal tract [9]. *E. histolytica* is an enteric extracellular protozoan that can attach and then destroy epithelial tissue. The most common manifestations accompanying this disease are bloody diarrhea, fever, abdominal pain, colitis, malaise, fatigue, flatulence, and weight loss [10]. It has 2 forms (trophozoites and cysts) in its life cycle. Infection occurs by the ingestion of water or food contaminated with cysts. In the small intestine, trophozoites excyst to develop and colonize the colonic region, and then adhere to the mucosal layer of the large intestines. *E. histolytica* causes intestinal and extra-intestinal infections [11]. In addition, some trophozoites may be excreted in stool outside the human host, but are not able to survive. The signaling pathways that lead to excystation or encystation are not clearly understood [12]. In extra-intestinal infections, *E. histolytica* parasites may penetrate the intestine wall to reach the liver through the portal circulation to form hepatic abscesses, which can be fatal if untreated. Abscesses may infect other organs, including the lungs and brain [8].

Previously, microscopic examinations were the only technique used in routine diagnostic laboratories to determine the presence of gastrointestinal parasites in stool specimens [13]. However, traditional diagnostic methods do not discriminate among the causative species of disease [14]. Since *E. dispar*, *E. bangladeshi*, and *E. moshkovskii* are morphologically identical to *E. histolytica*, molecular techniques have been used to facilitate the identification of *E. histolytica* at the genotype level [8]. Generally, polymerase chain reaction (PCR)-based techniques have higher sensitivity and specificity than microscopic tests [15]. Many studies have widely targeted unique regions of the small subunit ribosomal RNA fragment to diagnose the parasite, as a high copy number provides increased sensitivity [16].

This study was undertaken to determine the prevalence of amoebiasis during a 6-year period in the study area (Thi-Qar Province, Iraq). It also aimed to compare 2 PCR-based techniques (nested and real-time PCR) to determine is more sensitive for the genomes of *E. histolytica* and *E. dispar*.

## MATERIALS AND METHODS

### Study area and sample collection

Thi-Qar is a large province in southern Iraq. It is located at 31°14'N 46°19'E. The total area is 12,900 km^2^ and it includes approximately 2 million people [14]. It shares internal boundaries with Basrah, Missan, Wassit, and Muthanna Provinces. The capital of the province is Nasiriyah. Thi-Qar Province has a short winter and a very long, hot summer with a decreasing rainfall rate throughout the year [17].

All medical information regarding amoebiasis in Thi-Qar Province for 6 years (from the beginning of January 2015 until the end of December 2020) was taken from patient records at the Public Health Department of the Thi-Qar Health Office, including sex, date, age, and residence area. Direct smears were used to diagnose amoebiasis patients. The molecular studies were conducted at 2 locations: Bint Al-Huda and Mohammed Al-Mosawy Hospitals, from the beginning of February 2020 to the end of October 2020. These hospitals treat children ≤ 12 years of age. Eighty stool samples were randomly obtained from patients with diarrhea, and then were examined (general stool examination) using microscopy to observe trophozoites and/or cysts in the stool. Each of the 80 samples was kept in a stool container in a refrigerator (-20°C).

### Genomic DNA extraction

Genomic DNA was extracted from the stool using a stool DNA extraction kit (Bioneer, Daejeon, Korea), according to the manufacturer’s protocol. DNA concentration and purity were examined with a Nanodrop spectrophotometer (Thermo Fisher, Waltham, MA, USA). Extracted DNA was stored at -20°C for use in PCR amplification [18].

### Nested polymerase chain reaction assay

Two successive runs with 2 primers (external and internal primers) are involved in the nested PCR technique. A target region within the product of the first run is amplified by the second primer [19]. The *18S rRNA* gene was amplified to identify *E. histolytica* and *E. dispar*, according to Khairnar & Parija [20], which included two steps. The extracted DNA was mixed with external primers (E-1 5´-TAA GAT GCA GAG CGA AA-3´ and E-2 5´- GTA CAA AGG GCA GGG ACG TA-3´) for the detection of the *Entamoeba* genus at 888 bp. The nested PCR primers were prepared by IDT Company (Ottawa, ON, Canada). The PCR master mix was prepared according to KAPA2G Robust HotStart Ready Mix (2× ) PCR kit (Kapa Biosystems, Cape Town, South Africa). The master mix of the first run prepared 4 µL of DNA template, 12.5 µL of master mix 2× , 1.25 µL of 10 pmol of each external primer, and 6 µL of nuclease-free water. The thermal reaction consisted of initial denaturation at 96°C for 2 minutes followed by 30 cycles at 92°C for 60 seconds (denaturation), 56°C for 60 seconds (annealing) and 72°C for 90 seconds (extension), and final extension at 72°C for 7 minutes. The second run used the same concentration as the first run, except that 4 µL of the first run product was added as a DNA template. Amplification was conducted using the following internal primers: EH-1 5´-AAG CAT TGT TTC TAG ATC TGA G-3´ and EH-2 5´- AAG AGG TCT AAC CGA AAT TAG-3´ to identify *E. histolytica* (439 bp), and ED-1 5´- TCT AAT TTC GAT TAG AAC TCT-3´and ED-2 5´- TCC CTA CCT ATT AGA CAT AGC-3´ to detect *E. dispar* (174 bp). The thermal conditions of the second-run reaction were the same as the first run except that the annealing was at 48°C. These products were passed onto an agarose gel (2%) containing 3 µL of ethidium bromide. In the electrophoresis room, the tray was fixed and filled with a tris-borate-ethylenediaminetetraacetic acid buffer. A ladder (5 µL) was added into the first well, and 10 µL of each PCR product was placed into other wells. The electric current was set at 100 V and 80 mA for 1 hour. Finally, the electrophoresis product bands were imaged with an ultraviolet transilluminator.

### Real-time polymerase chain reaction assay

The *18S rRNA* gene was used to diagnose *E. histolytica* and *E. dispar*. Real-time PCR was carried out using the reverse primer (Ehd-88R 5´-GCGGACGGCTCATTATAACA-3´) and TaqMan probes for *E. histolytica*: histolytica-96 T; FAM5´ UCAUUGAAUGAAUUGGCCAUUU 3´-BHQ1 and *E. dispar*: dispar-96 T; HEX-5´ UUACUUACAUAAAUU GGCCACUUUG 3´-BHQ1 [21]. In real-time PCR, each species was visualized with different-colored filters. The 2× Kapa Probe qPCR Master Mix kit (Kapa Biosystems) used in the real-time quantitative PCR amplification and the master mix were prepared according to the manufacturer’s instructions, using 3 µL of genomic DNA, 12.5 µL of master mix, 1.5 µL of Ehd-88R primer, 1 µL of each of the histolytica-96 T and dispar-96 T probes, and 6 µL of nuclease-free water. The thermocycler conditions included an initial denaturation step at 95°C for 2 minutes, then followed by 40 cycles of denaturation and annealing/extension at 95°C and 60°C for 15 seconds and 30 seconds, respectively.

### Statistical analysis

In this study, the chi-square test was used to analyze the data in SPSS version 25 (IBM Corp., Armonk, NY, USA). The significance level was set at p-value≤ 0.05.

### Ethics statement

The protocol of the current study was received and approved by the Management of the Public Health Department of the Thi-Qar Health Office to analyze anonymized epidemiological information on participating patients. Oral consent was also obtained from participants’ parents for collecting and examining the stool.

## RESULTS

### Epidemiology

**Figure 1.**
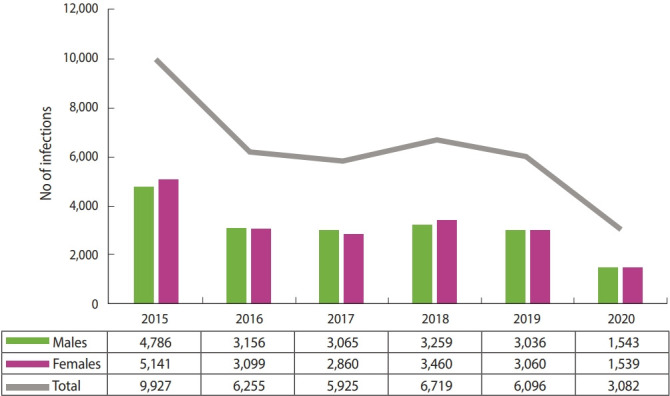
Amoebiasis infections distributed according to sex and year. Values are presented as number.

During the 6-year study period (from the beginning of January 2015 to the end of December 2020), a total of 38,004 (11.1%) out of 341,554 intestinal parasitic infections were classified as amoebiasis. Patients’ age ranged from < 1 year to 60 years. Of the 38,004 patients, 18,845 (49.6%) and 19,159 (50.4%) were males and females, respectively (Figure 1). No significant difference according to sex was found (p= 0.107). A significant difference (p < 0.01) in the percentage of amoebiasis among all intestinal parasitic infections was found between 2015 to 2020, but the overall trend over time showed oscillations (26.1, 16.5, 15.6, 17.7, 16, and 8.1%, respectively).

Table 1 shows that the highest proportion of amoebiasis cases was found in the age group of 5-14 years (27.3%), while the lowest proportion (9.0%) was recorded in the < 1-year age group. Statistically significant differences were found among age groups (p< 0.01), and the infection numbers decreased with increasing age.

**Figure 2.**
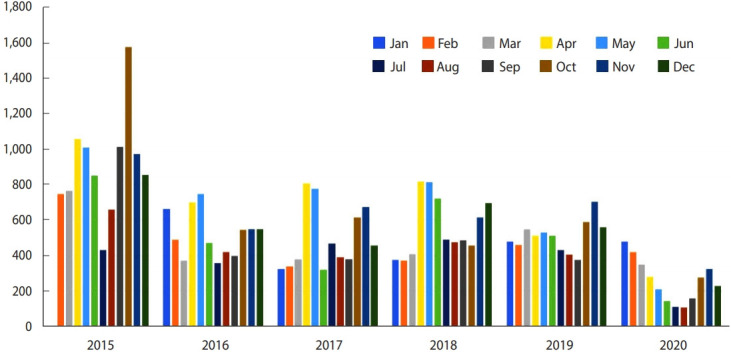
The distribution of amoebiasis according to month and year.

The majority (69.4%) of infected patients with amoebiasis resided in rural areas, whereas 30.6% were recorded in urban areas. This difference was statistically significant (p< 0.01). A statistically significant difference was also observed in the month of diagnosis (p≤ 0.05) (Figure 2).

### Molecular characterization

In nested PCR, the first-run findings showed that 42 (52.5%) out of 80 samples were positive for the *18S rRNA* gene (888 bp) of *Entamoeba* spp. The 80 samples were divided into 2 equal groups (one was contained trophozoites only, whereas the other included trophozoites and/or cysts); in these 2 groups, 10 samples and 32 samples were positive, respectively. Briefly, the samples that contained cysts were considered suitable for molecular examinations.

**Figure 3.**
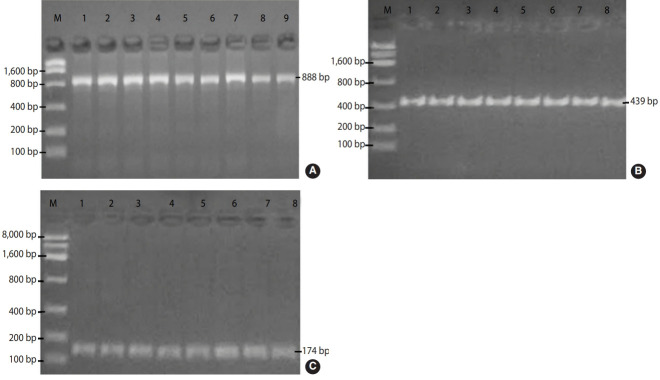
Images showing the detection of *Entamoeba* spp. (A), *E. histolytica* (B), and *E. dispar* (C) in amplified stools by nested polymerase chain reaction. Electrophoresis showed positive samples of *18S rRNA* (M: marker [100-8,000 bp]), (A) lanes 1-9 positive for *Entamoeba* spp. (first run) at 888 bp, (B) and (C) lanes 1-8 positive for *E. histolytica* and *E. dispar* bands at 439 and 174 bp, respectively.

Of the 42 samples of the second-run products that were electrophoresed, 25 (31.3%) were positive for *E. histolytica* (a 439-bp fragment) and 14 (17.5%) were positive for *E. dispar* (a 174-bp fragment) (Figure 3). Three (3.8%) were classified as Entamoeba spp. (Table 2).

**Figure 4.**
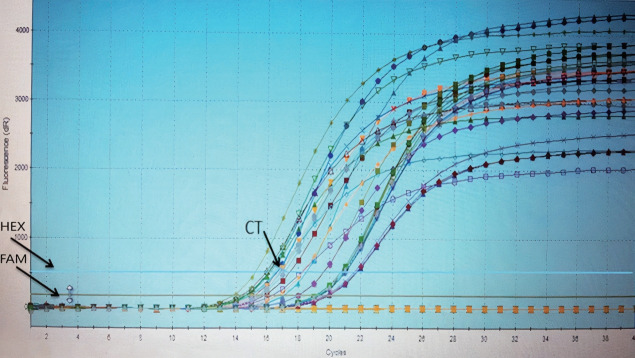
Graph showing the amplification products and the detection limits of the *18S rRNA* gene in *Entamoeba* spp. (*E. histolytica* and *E. dispar*) using real-time polymerase chain reaction. HEX, hexachlorofluorescein; FAM, fluorescent reporters 6-carboxyfluorescein; CT, cycle threshold.

From the 42 positive samples of *18S rRNA* gene, *E. histolytica* and *E. dispar* were detected in 28 (35.0%) and 15 (18.8%) samples by real-time PCR, respectively (Figure 4). No statistically significant differences were found between *E. histolytica* and *E. dispar* with the 2 techniques that were used.

## DISCUSSION

Epidemiological studies on the prevalence of parasitic intestinal infections in different areas have usually aimed to identify communities at risk and diseases that pose risks to human populations, making it necessary to study infections that threaten human health throughout the world [22]. Several environmental, biological, behavioral, socioeconomic, and health-related factors affect parasitic infections directly or indirectly. The quality of city or village infrastructure, income, occupation, and education level are also important aspects that affect the spread of infection, disease transmission, and mortality [23].

In this study, there were 38,004 enrolled patients with amoebiasis in the 6-year study period (49.6% males and 50.4% females), corresponding to an even distribution by sex. This result aligns with those reported by Al-Damerchi & Al-Ebrahimi [24] and Hamza et al. [25] in Iraq. The year 2020 had the lowest infection rate during the study period, which may have been due to the coronavirus disease 2019 (COVID-19) pandemic in Iraq, which led people to fear hospitals and health centers. In the current study, the infection rates differed by age, and were highest in the 5-14 age group. Variation in intestinal parasitic infections by age is expected, as daily activities and behavioral habits play an important role in determining the time and type of exposure to the infective stage of the parasite [26]. Our results agree with the findings of several studies, such as those of Saida [22]; Al-Taei [27]; Al-Saqur et al. [28] in Iraq; Nath et al. [7] in India and Al-Dalabeeh et al. [8] in Jordan. The study recorded a higher proportion of infections in rural areas than in urban areas, which is compatible with previous studies, such as those conducted by Nath et al. [7]; Al-Damerchi & Al-Ebrahimi [24], and Al-Dalabeeh et al. [8]. The proportion of infections by month fluctuated during the 6-year study period, but environmental conditions such as the temperature, drinking water pollution, health services, and nutritional behavior, which are generally considered to vary over time, affect the incidence of parasitic infections [27]. These results are similar to those of Nath et al. [7] and Saida [22].

Microscopic examinations of *E. histolytica* are often inaccurate and unreliable, especially in samples containing morphologically identical species such as *E. dispar*, *E. bangladeshi*, and *E. moshkovskii*, so molecular tools are useful for the specific identification of Entamoeba spp. [8,29]. In this study, among the 80 stool samples that were microscopically identified as being positive for *Entamoeba*, 42 were positive for the *18S rRNA* gene. Of these samples, 25 and 14 were positive for *E. histolytica* and *E. dispar* with nested PCR, respectively, whereas 28 and 15 were found to be positive using real-time PCR. This result aligns with those of other studies, such as those conducted by Mohammed et al. [30], Al-Saqur et al. [28], and Faqe Mahmood & Mustafa [18]. However, the low results for molecular identification may be due to the lysis of trophozoites resulting from sample storage or due to the presence of other *Entamoeba* spp. that are morphologically similar to *E. histolytica* and *E. dispar* [4,20].

In conclusion, epidemiological research provides insights into the incidence and prevalence of a disease according to factors such as sex, endemic areas, temperature, economic activity, and the health environment. This study analyzed amoebiasis, which is considered a health burden and uncontrolled disease in Thi-Qar Province, especially in low-hygienic and poor regions. Although all the reported cases in this study were treated with anti-amoebic drugs, it is thought that many cases of *E. dispar* infection were treated unnecessarily. This study suggests the need to conduct molecular identification before treatment, and found that both nested and real-time PCR were beneficial for diagnosing *Entamoeba* spp.

## Figures and Tables

**Table 1.** Demographic data of amoebiasis in relation to sex, age, and residential area during the 6-year study period

Characteristics	n (%)
Sex	
Male	18,845 (49.6)
Female	19,159 (50.4)
Age (yr)	
<1	3,416 (9.0)
1-4	8,536 (22.5)
5-14	10,383 (27.3)
15-45	10,129 (26.6)
>45	5,540 (14.6)
Residential area	
Urban area	11,636 (30.6)
Rural area	26,368 (69.4)

**Table 2.** Comparison of results of nested PCR and real-time PCR of *E. histolytica* and *E. dispar*

*Entamoeba* spp.	Real-time PCR			Nested PCR		
	Trophozoites	Cysts ± trophozoites	Total, n (%)	Trophozoites	Cysts ± trophozoites	Total, n (%)
*E. histolytica*	5	20	25 (31.3)	7	21	28 (35.0)
*E. dispar*	4	10	14 (17.5)	5	10	15 (18.8)
Other	1	2	3 (3.8)	-	-	-

PCR, polymerase chain reaction.
